# Supplementary material for: Therapeutic modulation of phagocytosis in glioblastoma can activate both innate and adaptive antitumour immunity
Source: Nat Commun. 2020 Mar 20;11:1508. doi: 10.1038/s41467-020-15129-8 (PMC7083893; doi:10.1038/s41467-020-15129-8)
Supplement: Supplementary file 1 — Supplementary Information [file 41467_2020_15129_MOESM1_ESM.pdf]

Therapeutic modulation of phagocytosis in glioblastoma can activate both innate and adaptive antitumour immunity

Von Roemeling et al.

SUPPLEMENTARY FIGURES

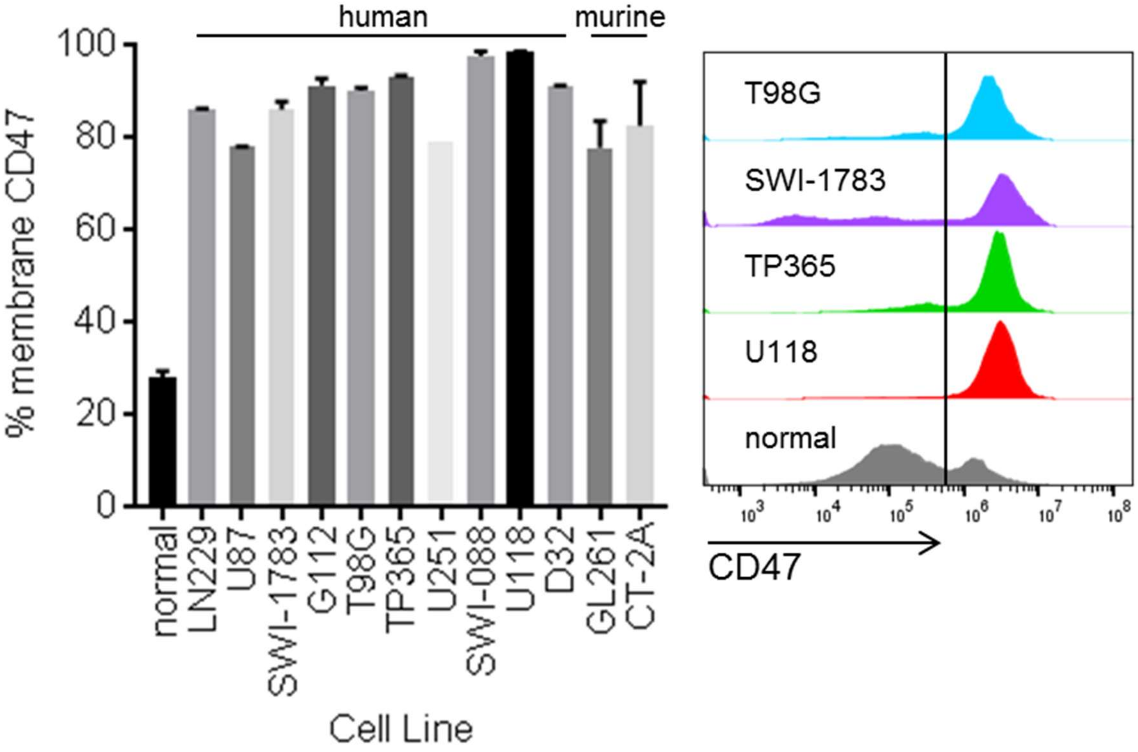

Supplementary Figure 1: CD47 is highly expressed in both human and mouse GBM cell lines. More than 80% of cells are positive for CD47 based on flow cytometry analysis. n=2, error bar = mean ± standard deviation. Normal: primary astrocytes isolated from normal brain tissue samples after temporal lobectomy for epilepsy surgery.

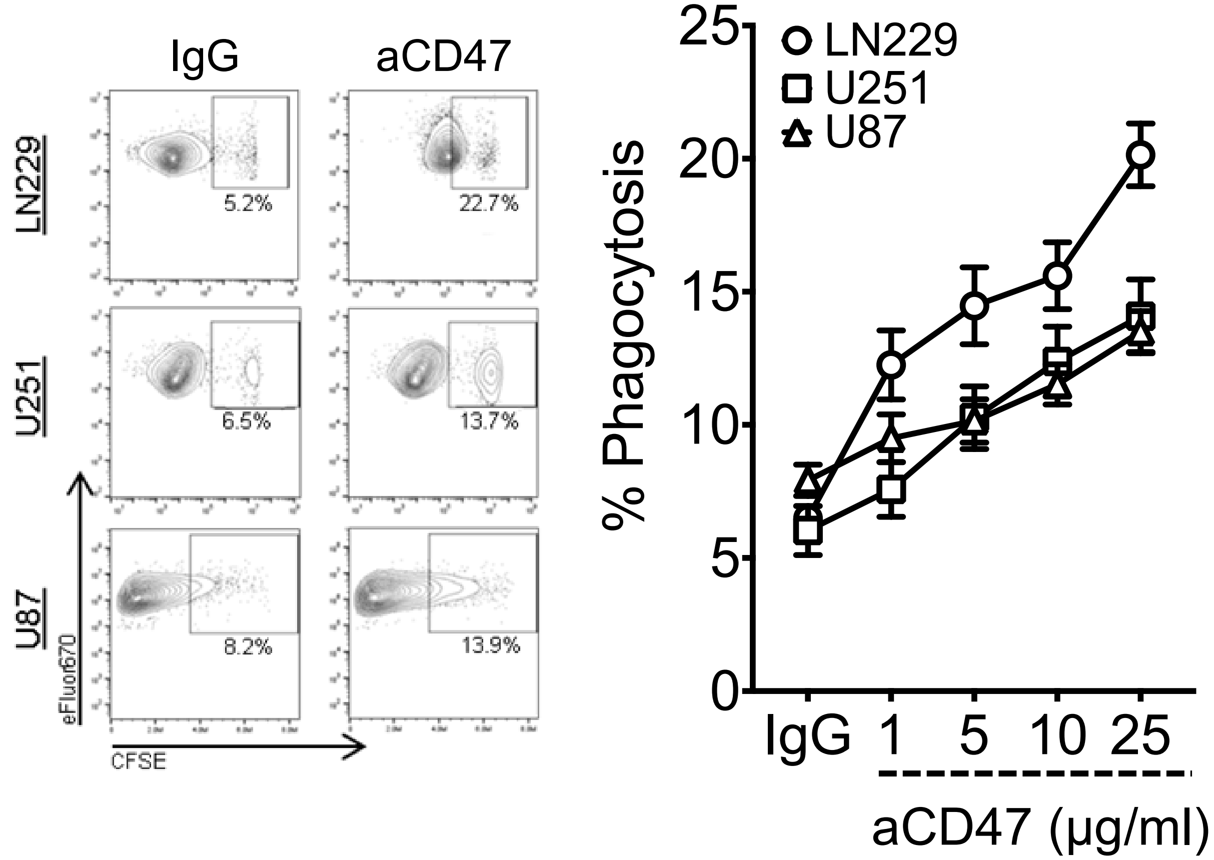

Supplementary Figure 2: Anti-CD47 antibody (aCD47, clone B6H12) treatment increased the phagocytosis of human GBM cells by human THP-1 cells. Representative results are shown in the left panel. n=6, error bar = mean ± standard deviation.

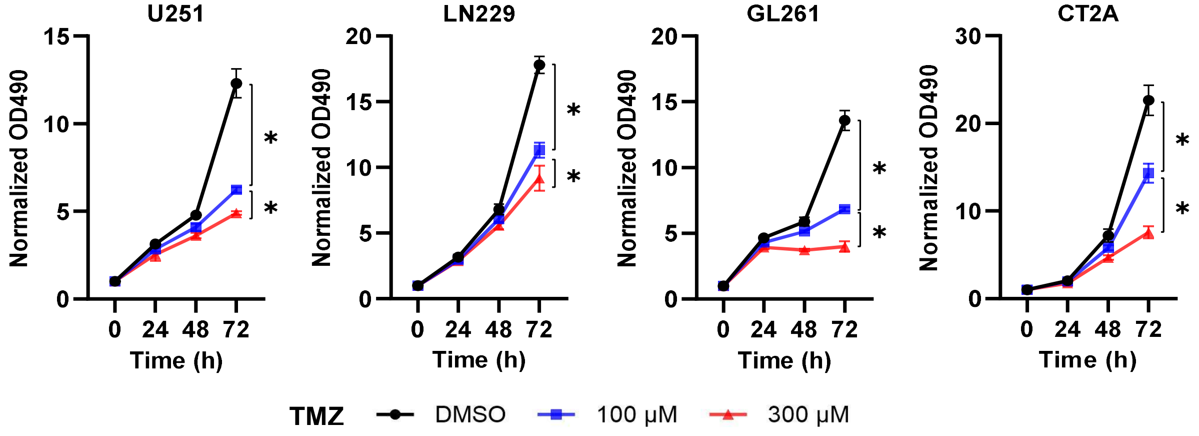

Supplementary Figure 3: Growth inhibitory effect of TMZ on glioma cells. n=3, error bar = mean ± standard deviation. *$p < 0.05$, unpaired Student's t test.

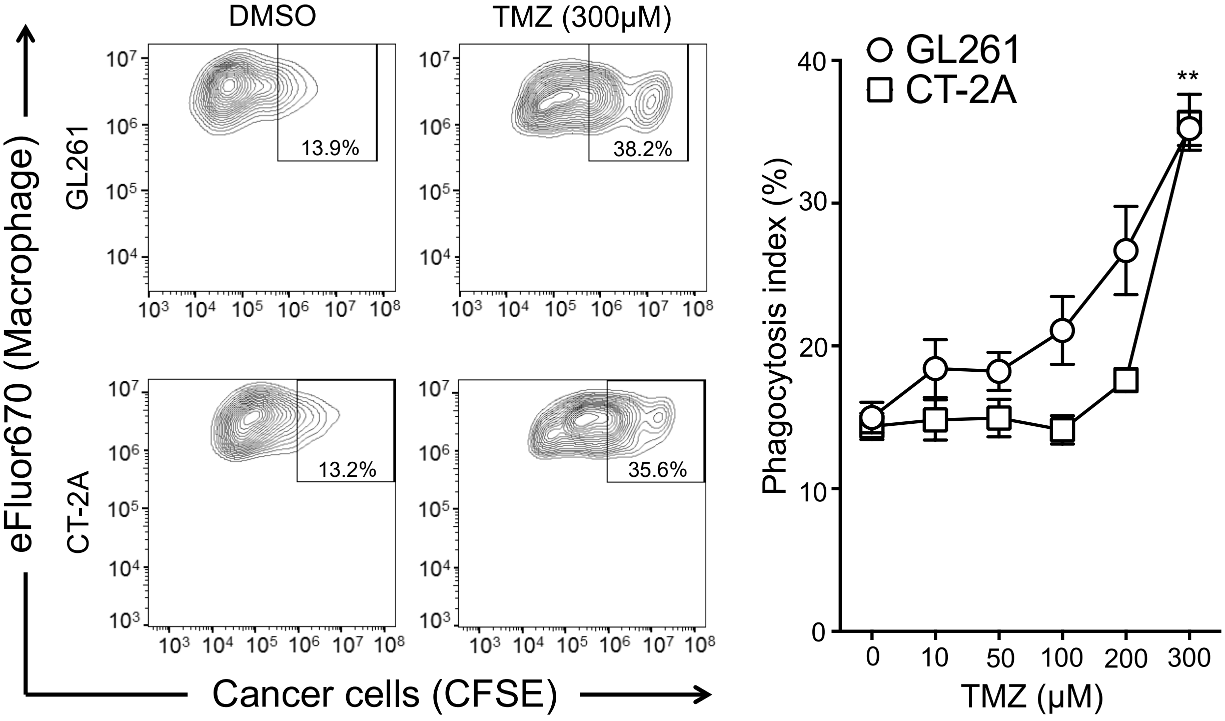

Supplementary Figure 4: TMZ treatment promotes phagocytosis of murine GBM cells by mouse bone marrow derived phagocytes. n=6, error bar = mean ± standard deviation. **p<0.01, compared to 0 μM, unpaired Student's t test.

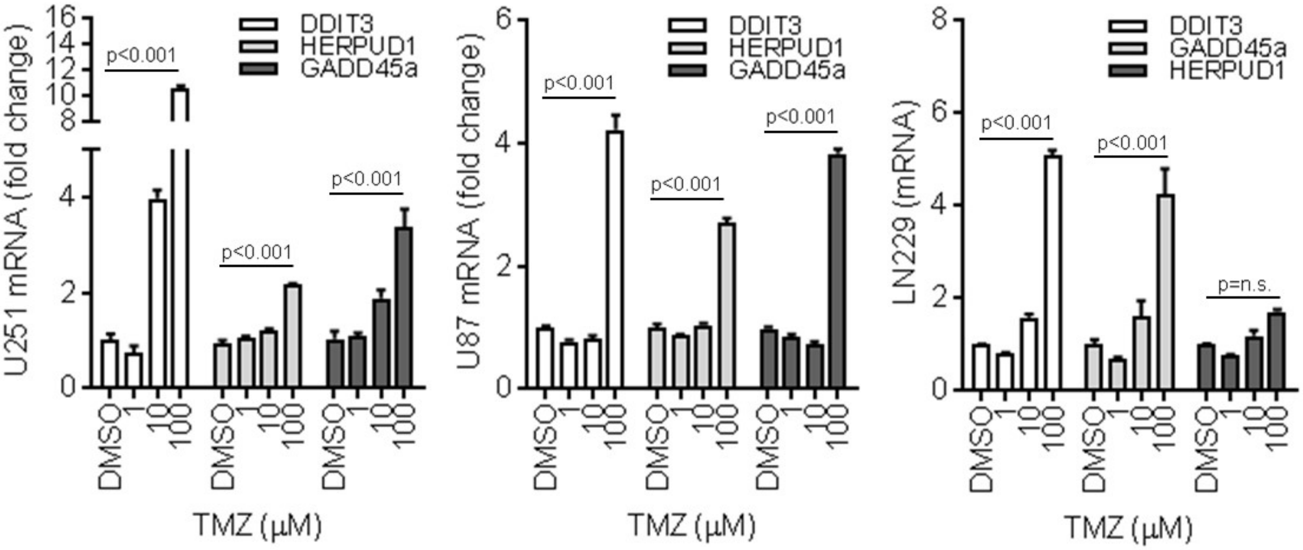

Supplementary Figure 5: TMZ treatment up-regulated the mRNA expression of ER response-associated targets DDIT3, HERPUD1 and GADD45α in human GBM cells. n=3, error bar = mean ± standard deviation, unpaired Student's t-test.

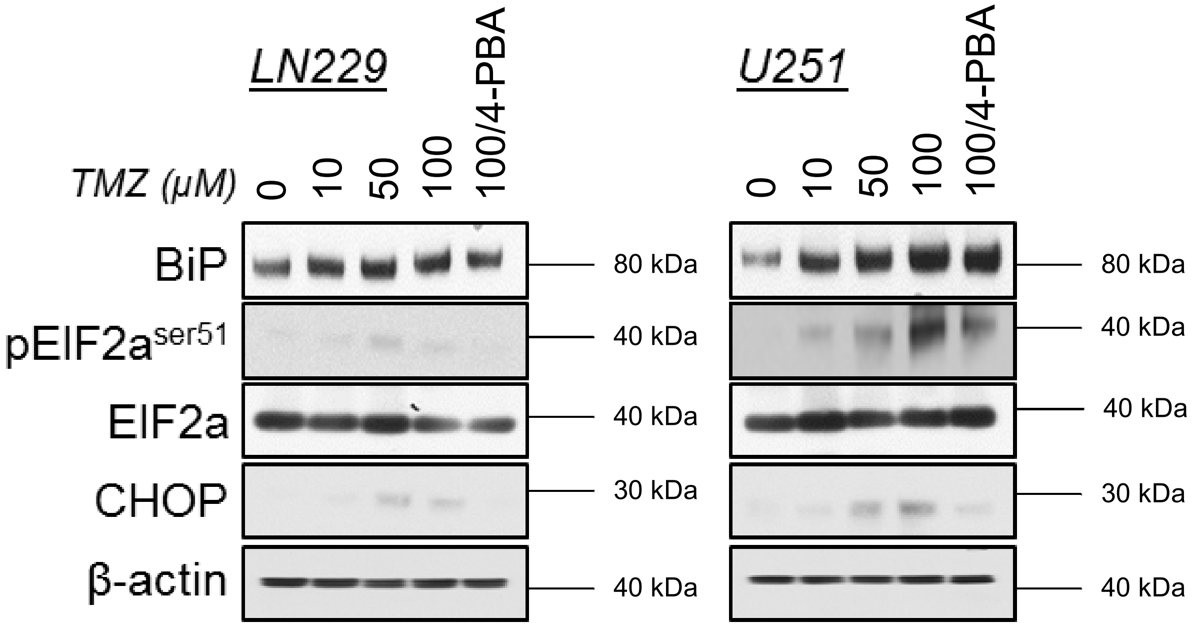

Supplementary Figure 6: Western blot showing TMZ treatment increased the expression levels of ER stress response-specific protein BiP, phospho-EIF2α and CHOP in mouse GBM cells, a response that is decreased by the addition of the ER stress inhibitor 4-PBA.

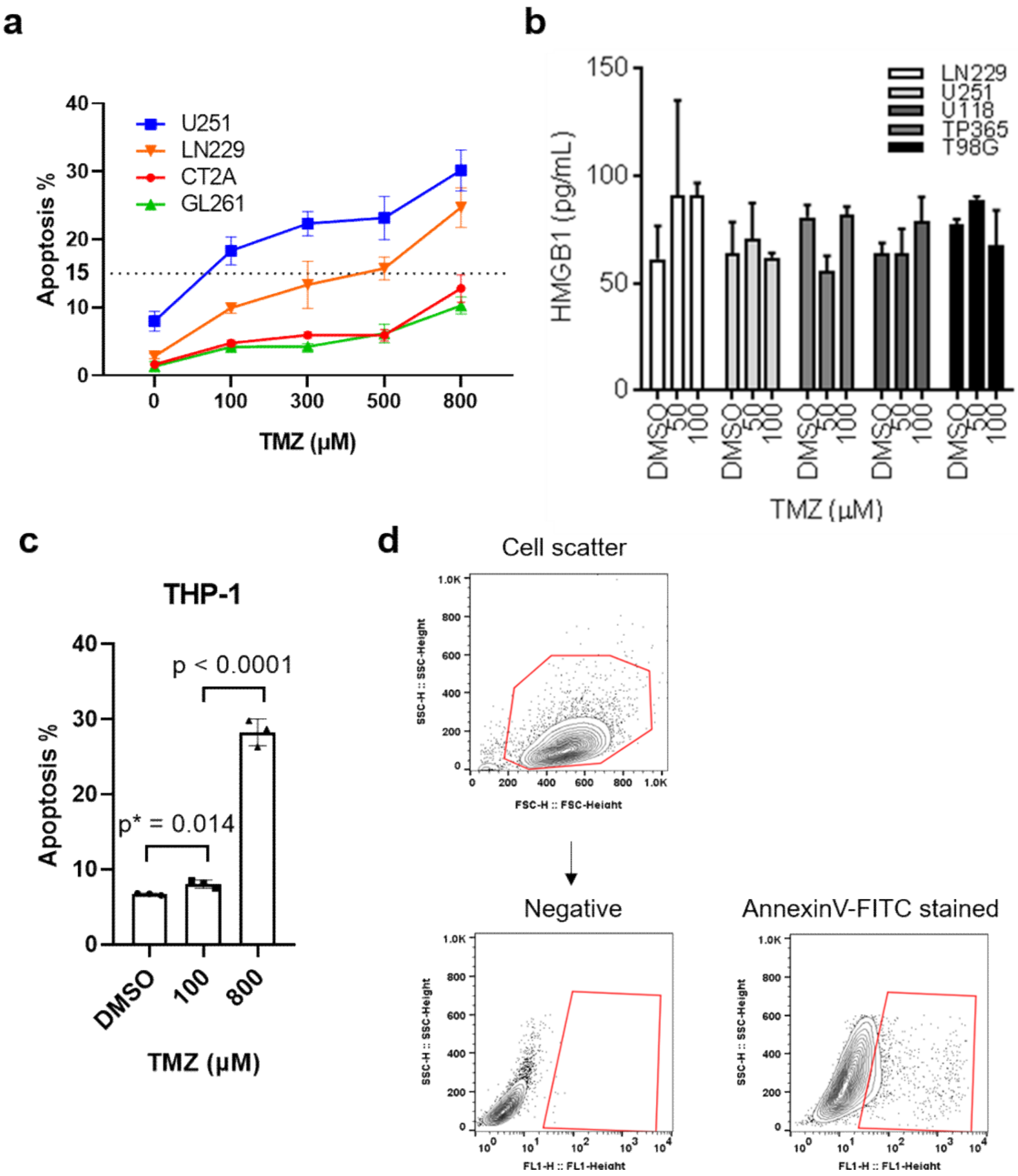

Supplementary Figure 7: TMZ induces apoptosis at high doses. a) TMZ treatment for 72 hours did not induce severe apoptotic cell death in mouse and human GBM cells until the dose exceeded 500 µM. n=3 b) TMZ treatment did not result in significant increase in the release of HMGB1 from human GBM cells. n=2, error bar = mean ± standard deviation. c) High dose TMZ treatment for 72 hours induced apoptosis in THP-1 cells. n=3, error bar = mean ± standard deviation. P-values were calculated by the student's t-test. d) Representative gating strategy to determine apoptotic cells by flow cytometry.

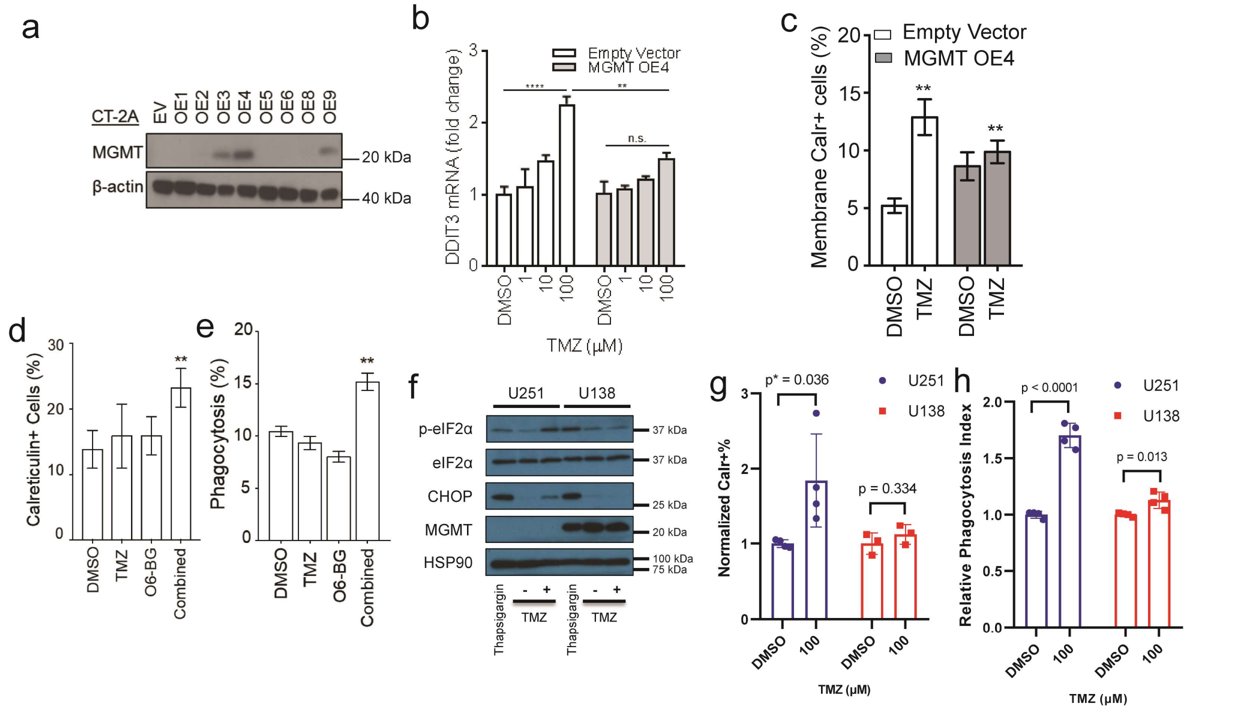

Supplementary Figure 8: TMZ-induced ER-stress depends on MGMT deficiency. a) Induced-expression of MGMT in murine GBM cells (CT-2A) significantly reduced TMZ-mediated upregulation of DDIT3 b), and the translocation of calreticulin (Calr) to the plasma membrane. n=3 c). The MGMT overexpressing clone (OE4) was selected for testing. n=6, error bar = mean ± standard deviation. **p<0.05, between empty vector and MGMT OE4 groups, unpaired Student's t test. ****<0.01, by one way ANOVA. d-e) Addition of O^6-benzylguanine (O6BG), a synthetic derivative of guanine and substrate competitor for MGMT methyltransferase activity restored TMZ-induced calreticulin translocation and phagocytosis response in murine MGMT overexpressing (OE4) GBM cells. n=6, error bar = mean ± standard deviation. **p<0.01, compared with TMZ alone, unpaired Student's t test. f) TMZ treatment upregulated of ER-stress markers of U251 cell line, but not of U138. g) TMZ treatment enhanced more calreticulin translocation (U251, n=4, U138, n=3) and h) phagocytosis of the U251 than U138 cells (n=4), error bar = mean ± standard deviation, unpaired Student's t test.

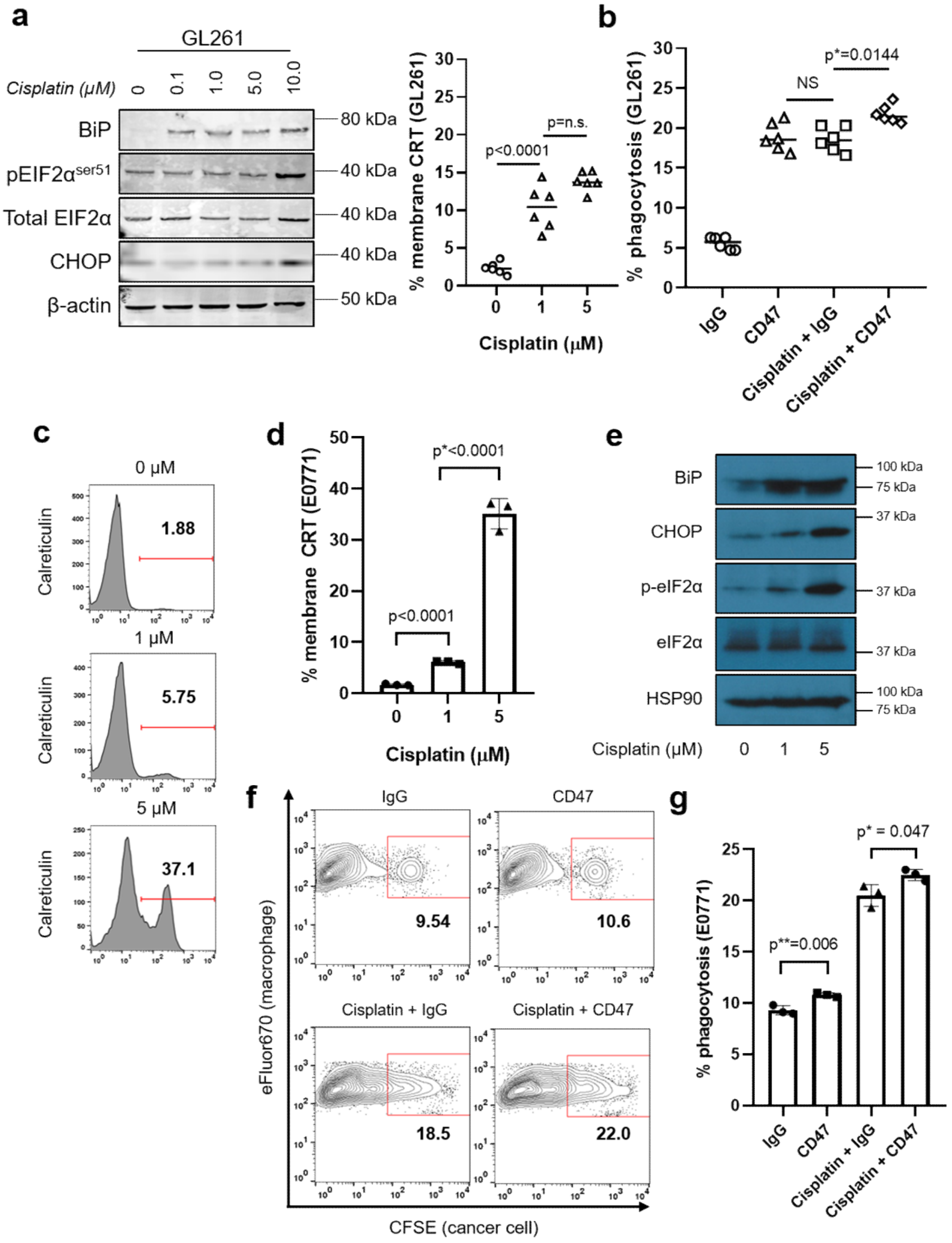
a
GL261
Cisplatin (μM)
0
0.1
1.0
5.0
10.0
BiP
pEIF2α ser51
Total EIF2α
CHOP
β-actin
80 kDa
40 kDa
40 kDa
40 kDa
50 kDa
% membrane CRT (GL261)
p<0.0001
p=n.s.
Cisplatin (μM)
b
% phagocytosis (GL261)
NS
p*=0.0144
IgG
CD47
Cisplatin + IgG
Cisplatin + CD47
c
0 μM
1.88
1 μM
5.75
5 μM
37.1
Calreticulin
d
% membrane CRT (E0771)
p<0.0001
p*<0.0001
Cisplatin (μM)
e
BiP
CHOP
p-eIF2α
eIF2α
HSP90
100 kDa
75 kDa
37 kDa
Cisplatin (μM)
f
IgG
CD47
Cisplatin + IgG
Cisplatin + CD47
9.54
10.6
18.5
22.0
eFluor670 (macrophage)
CFSE (cancer cell)
g
% phagocytosis (E0771)
p**=0.006
p* = 0.047

Supplementary Figure 9: Cisplatin induces ER-stress in GBM and breast cancer cells. a) Western blot of ER-stress markers (left) and calreticulin translocation (right) of GL261 cell line after 48 hours of cisplatin treatment. n=6. b) Phagocytosis of GL261 by BM cells was enhanced by cisplatin (1 μM) and/or anti-CD47 treatment. n=6. c-d) Cisplatin treatment for 48 hours induced calreticulin translocation. n=3. e) Cisplatin treatment induced ER-stress markers upregulation of breast cancer cell line E0771. f,g) Phagocytosis of E0771 by BM cells was enhanced by cisplatin (1 μM) and/or anti-CD47 treatment. n=3 error bar = mean ± standard deviation, unpaired Student’s t test.

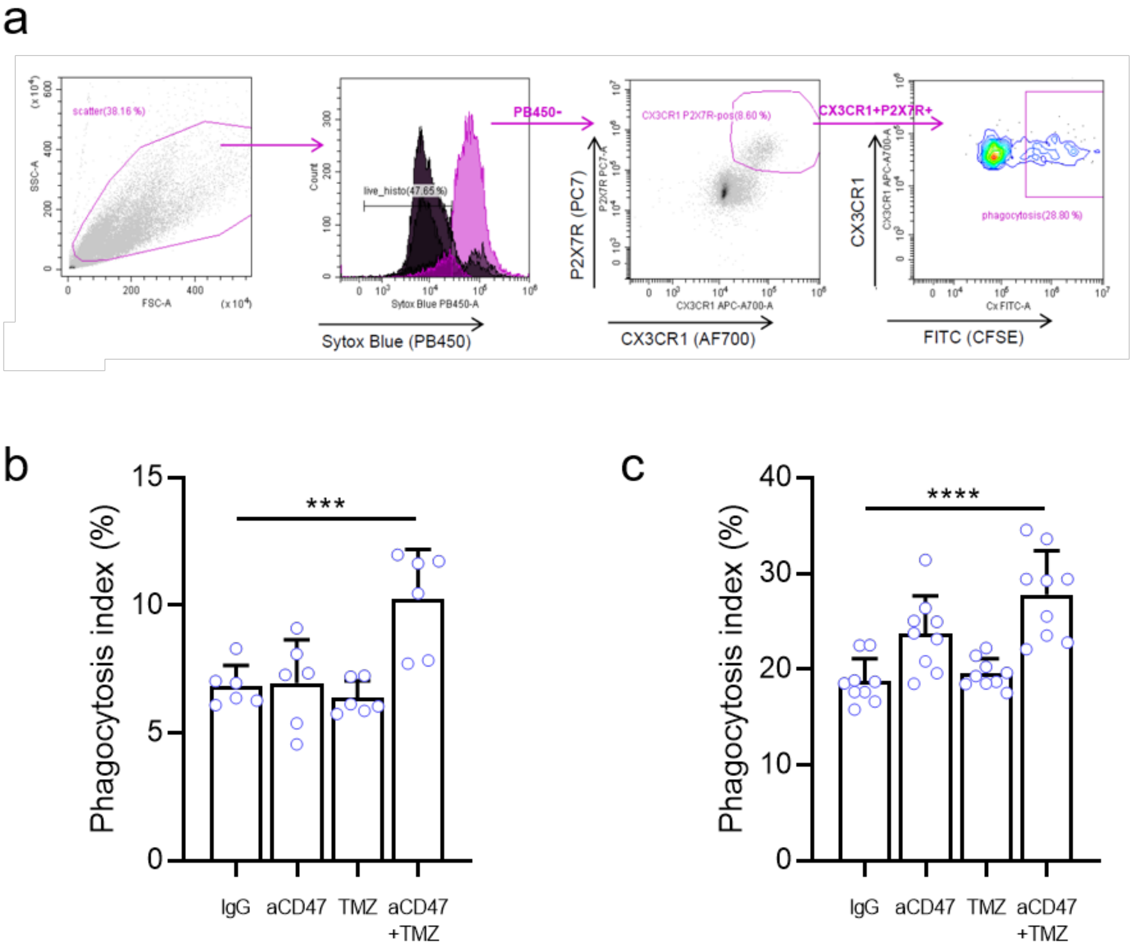

Supplementary Figure 10: Combined TMZ and anti-CD47 treatment promotes phagocytosis of murine GBM cells by mouse primary microglia. a) Gating strategy for isolating primary microglia. b) Phagocytosis assay in CT-2A (n=6) and c) GL261 (n=9) cells. Error bar = mean ± standard deviation. P-value obtained using one-way ANOVA adjusted for multiple comparisons using Tukey's method.

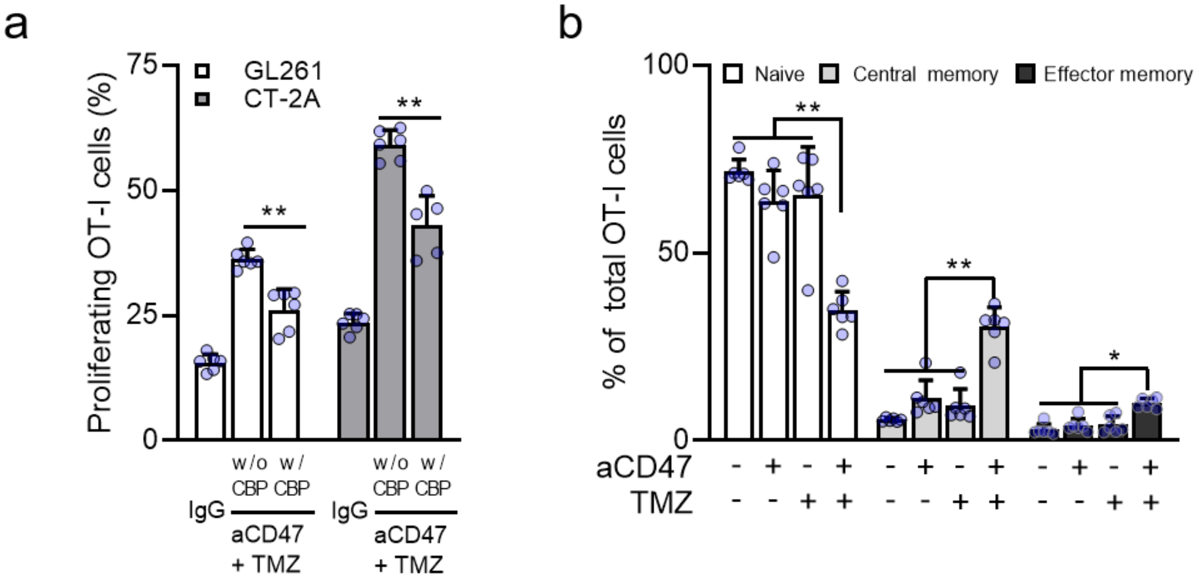

Supplementary Figure 11: Combined anti-CD47 and TMZ treatment enhances T cell priming. a) Blocking of Calreticulin with a blocking peptide (CBP) significantly inhibited the cross-priming of OT-I T cells by BM APCs in the setting of anti-CD47 antibody and TMZ treatment for murine cOVA-transduced GBM cells. n=6, error bar = mean ± standard deviation. **p<0.01, unpaired Student's t test. b) Enhanced cross-priming effect was observed with combination anti-CD47 antibody and TMZ treatment (in GL261-cOVA cells) resulted in a shift of naïve OT-I T cells towards memory phenotypes. n=6, error bar = mean ± standard deviation. **p<0.01, compared with monotherapy groups, unpaired Student's t test.

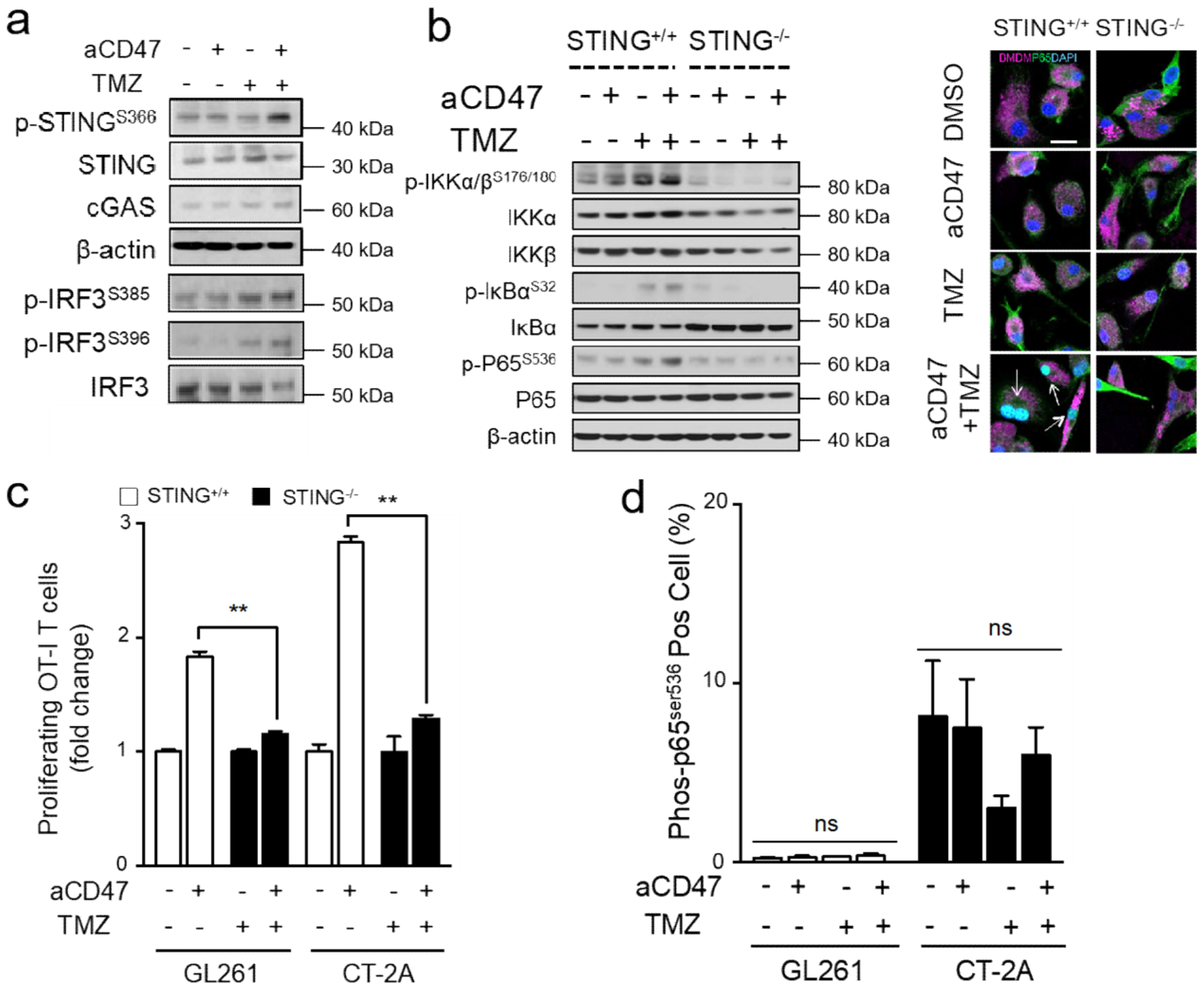

Supplementary Figure 12: Combined anti-CD47 antibody and TMZ treatment against mouse GBM cells activates cGAS-STING cytosolic DNA sensing pathway in murine BM APCs. a) Western blot of CT-2A cells after treatment. b) Phosphorylation and nuclear translocation of p65 subunit of NF-κB in the setting of combined anti-CD47 antibody and TMZ treatment in CT-2A cells are diminished in STING-deficient Tmem173^{gt} BM APcs. c) Deficiency of STING abrogated the ability of APCs to cross-prime T cells after incubating with anti-cD47 antibody and TMZ treated cOVA-transduced murine GBM cells. n=5. d) Phosphorylation of p65 is minimal and non-significant in tumour cells with combination treatment. n=4, error bar = mean ± standard deviation. **p<0.01, for indicated comparisons, ns= not significant.

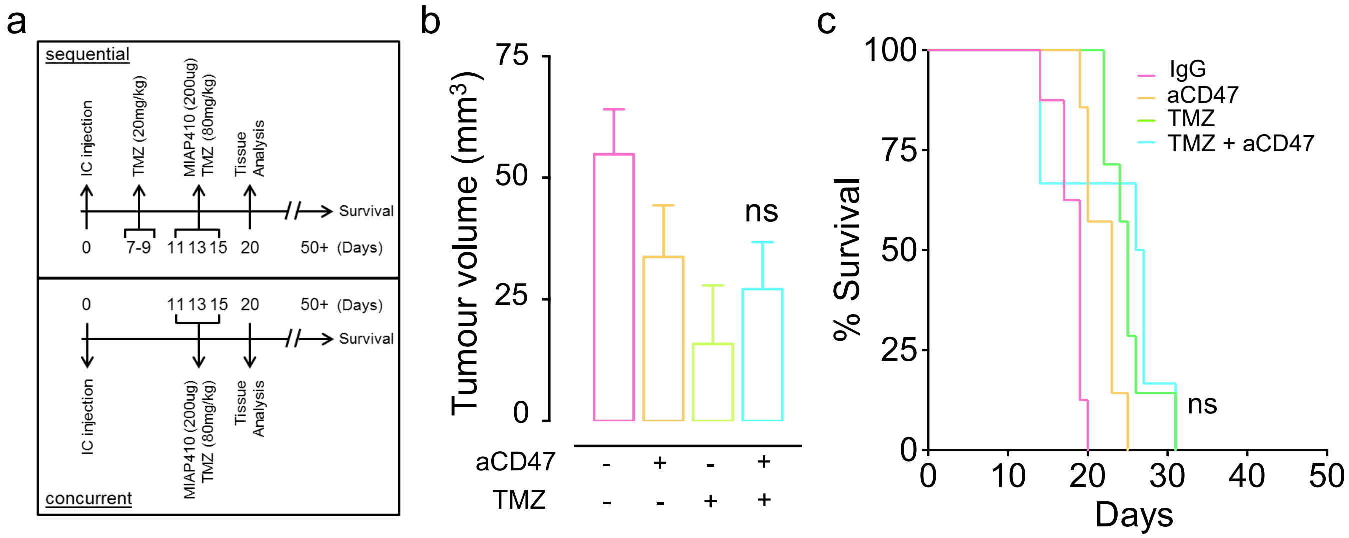

Supplementary Figure 13: Effect of combined anti-CD47 and TMZ treatment on murine GBM models. a) Treatment schema for concurrent and sequential combination therapy with anti-CD47 antibody and TMZ treatment of GL261 model. Concurrent anti-CD47 antibody and TMZ treatment did not result in improved tumour growth inhibition (n=5) b) or animal survival (n=8 in control group, n=7 in other groups) c) against TMZ or anti-CD47 antibody treatment alone, error bar = mean ± standard deviation, ns = not significant. Unpaired Student’s t test for b), Log-rank test for c).

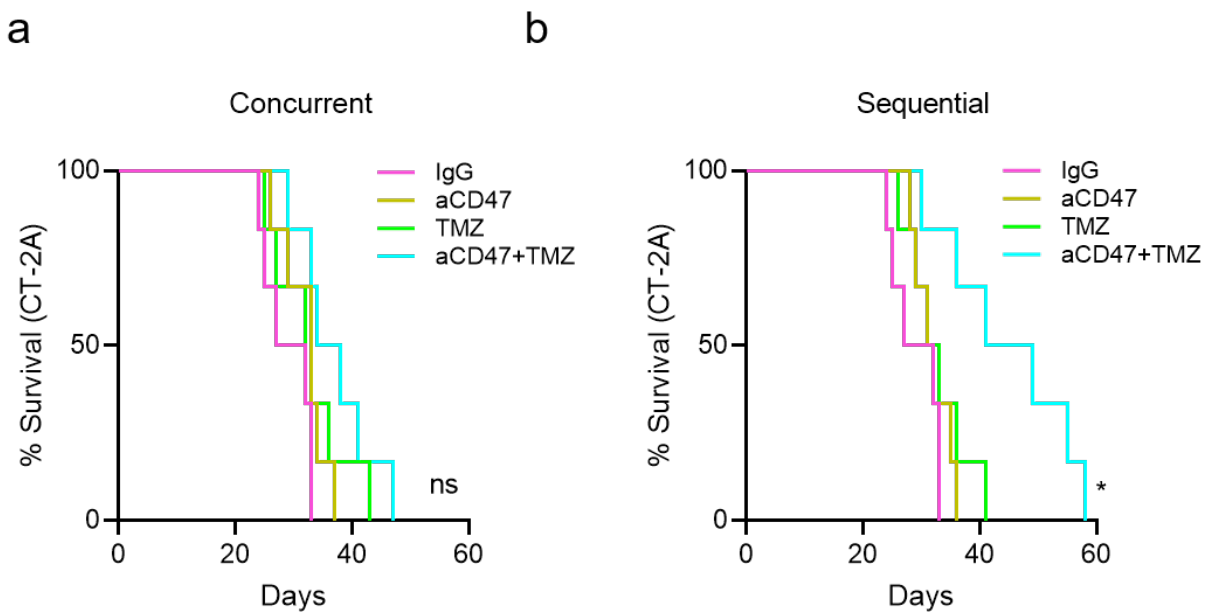

Supplementary Figure 14: Survival plot of CT-2A tumour bearing mice treated with concurrent (a) or sequential (b) regimen of combined anti-CD47 antibody and TMZ therapy. n=6, ns = not significant, **p<0.01, Log-rank test.

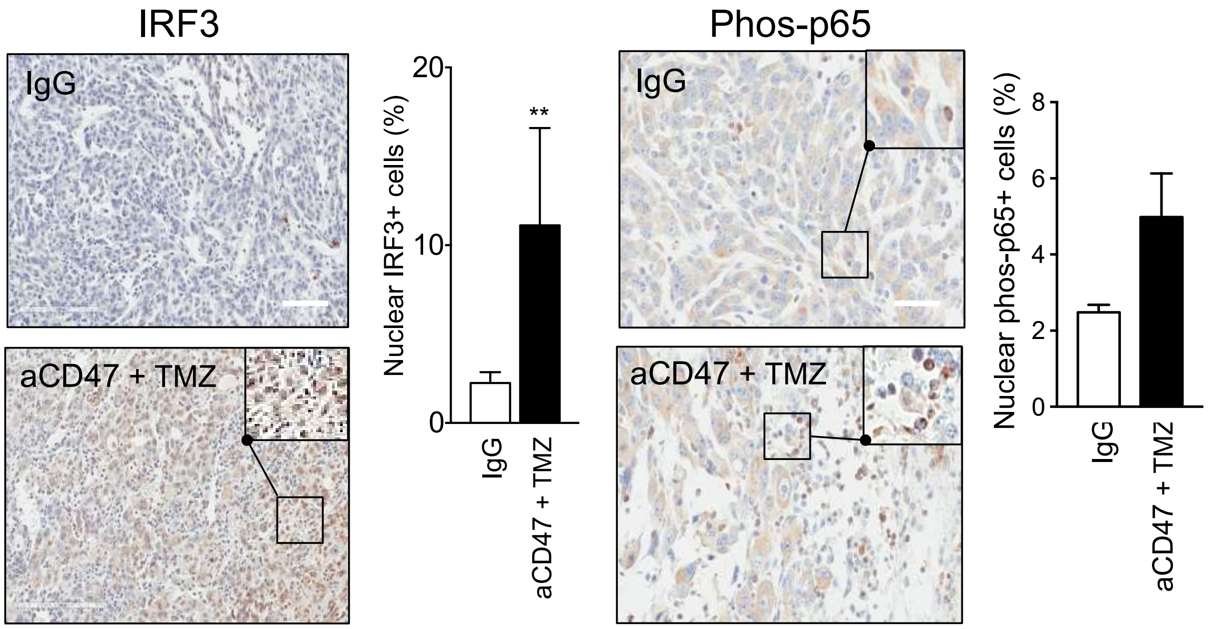

Supplementary Figure 15: Combined anti-CD47 antibody and TMZ treatment increased the level of nuclear IRF3 (n=5) and phosphorylated p65 (n=6 in the control group, n=5 in the aCD47+TMZ group) in vivo. Scale bar = 100μm. Bar graph = quantified data of 10 representative areas from tumour samples. Error bar = mean ± standard deviation. **p<0.01, Student's t test.

Supplementary Figure 16: Uncropped original gel images of western blots.

Fig 2F

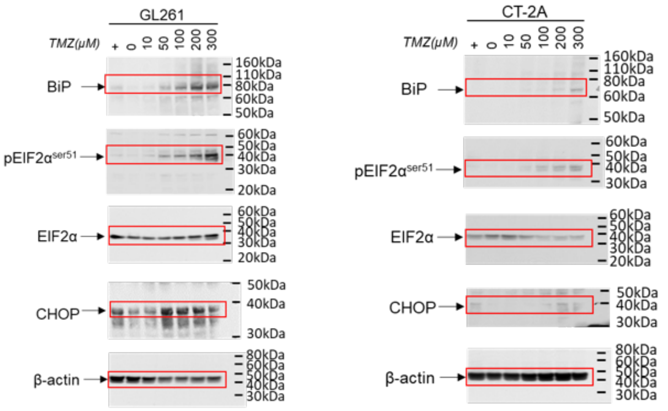

Fig 4b

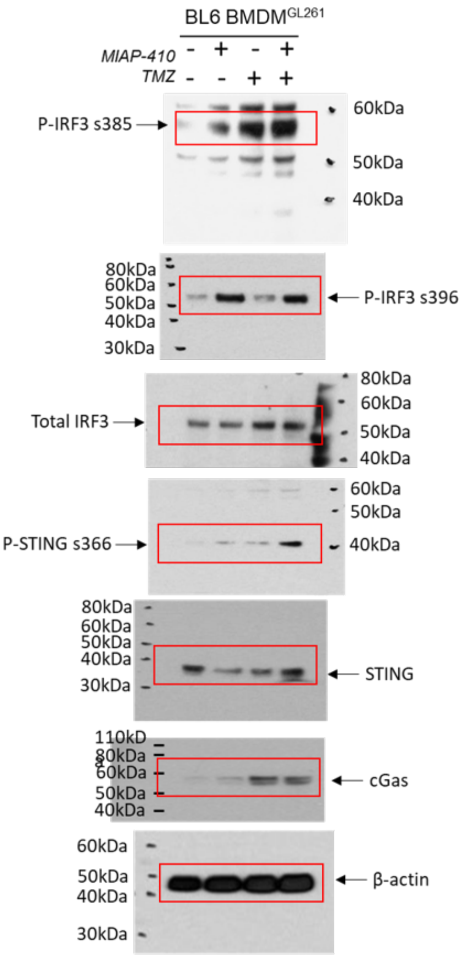

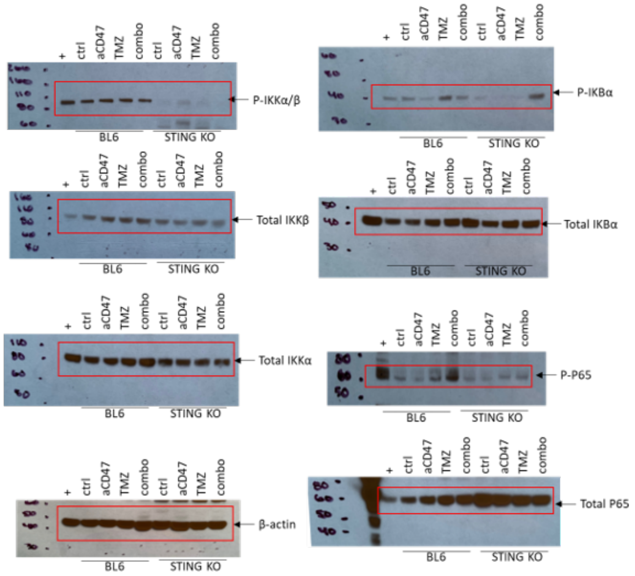

Supp. Fig 6

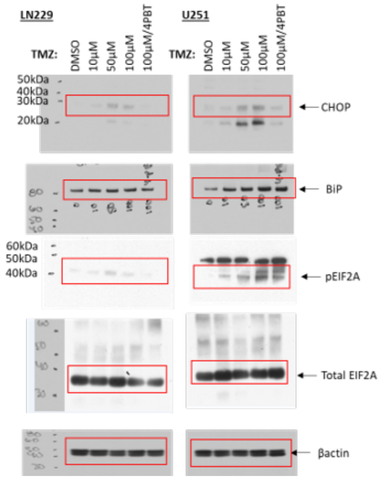

Supplementary Figure 16 (continued): Uncropped original gel images of western blots.

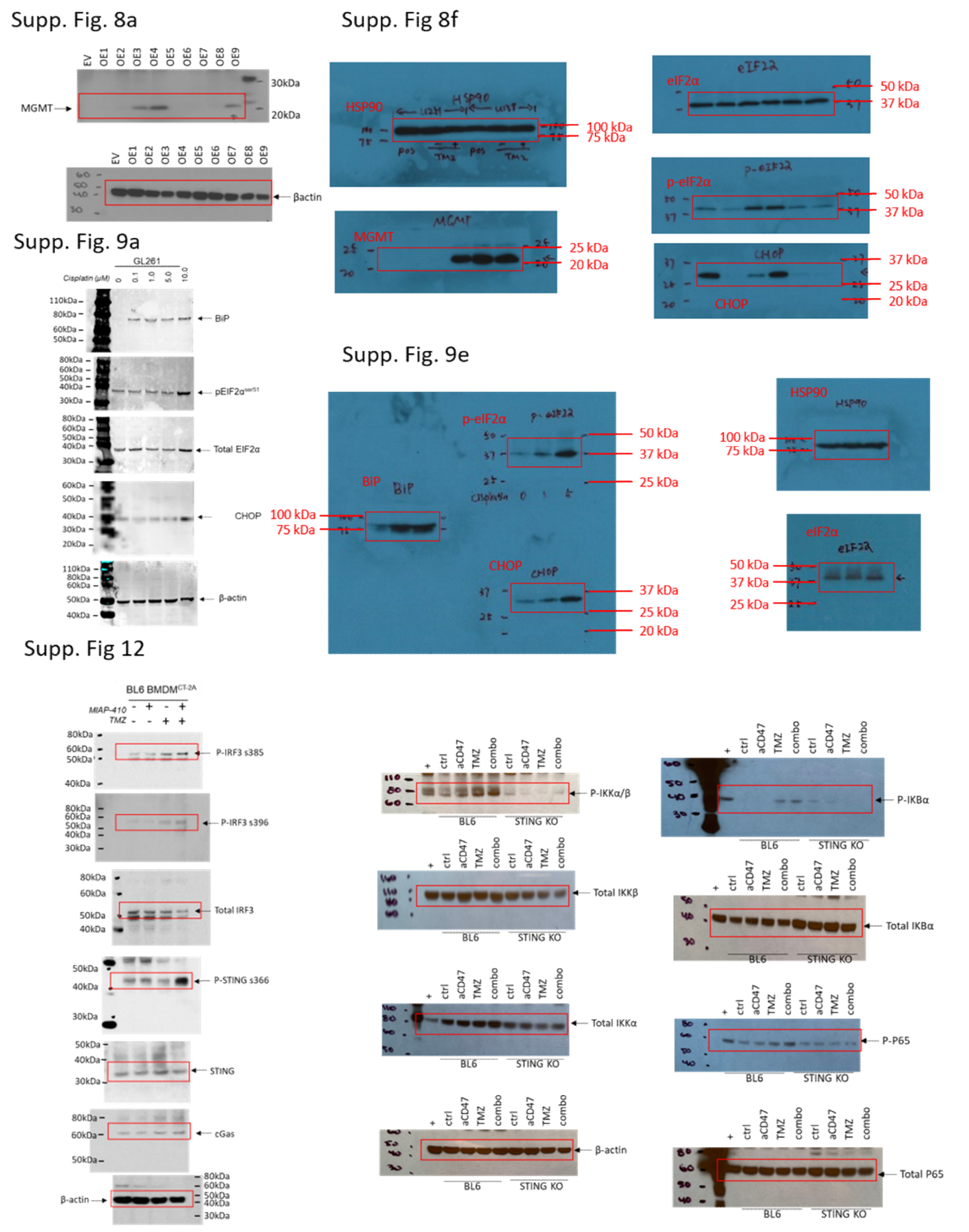

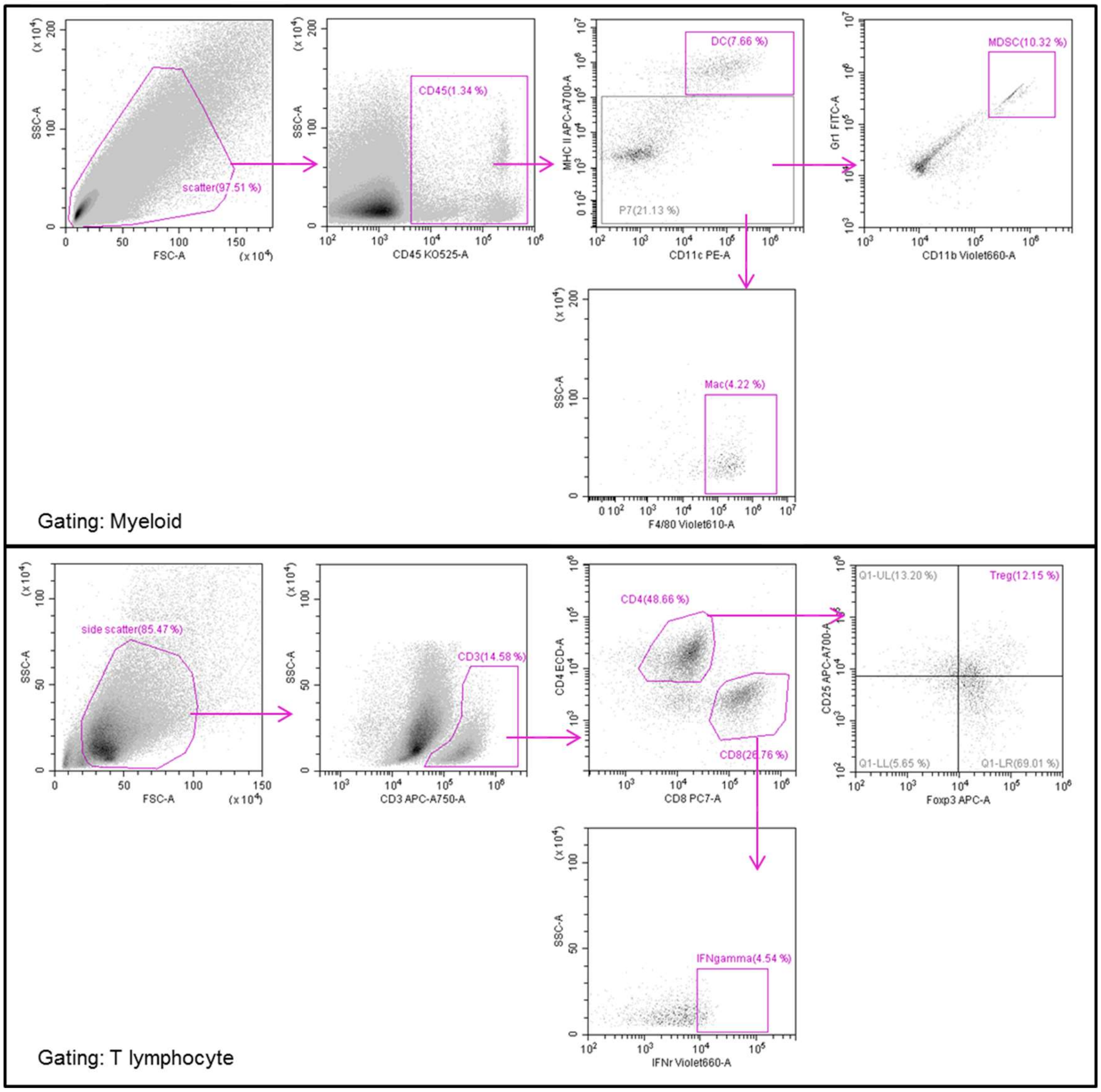

Supplementary Figure 17: Flow cytometry gating strategy for profiling myeloid and lymphoid cell populations.

Supplementary Figure 18: Gating strategies for Flow cytometry experiments.

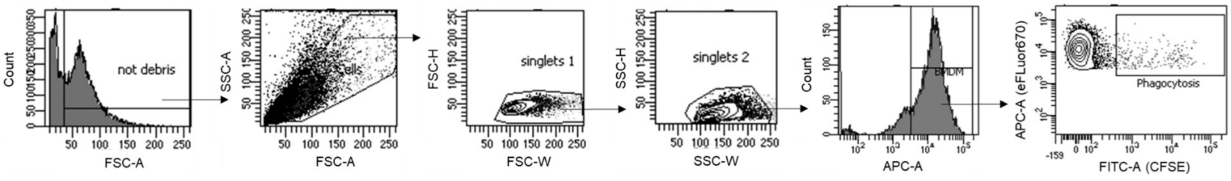

Gating strategy used for phagocytosis. Gating strategy to identify fraction of BMDM actively phagocytosing tumor cells following co-culture in response to drug treatment used in figures 1b, 2d, 2g, 3a-c and supplemental figures 2, 4, 8e, 8h, 9b, 9f-g.

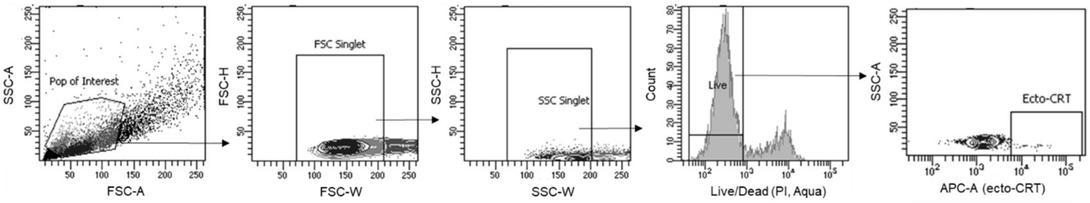

Gating strategy used for calreticulin translocation. Gating strategy to identify fraction of tumor cells expressing cell surface calreticulin in response to drug treatment used in figures 2a-c, 2g, and supplemental figures 8c-d, 8g, 9a, and 9d.

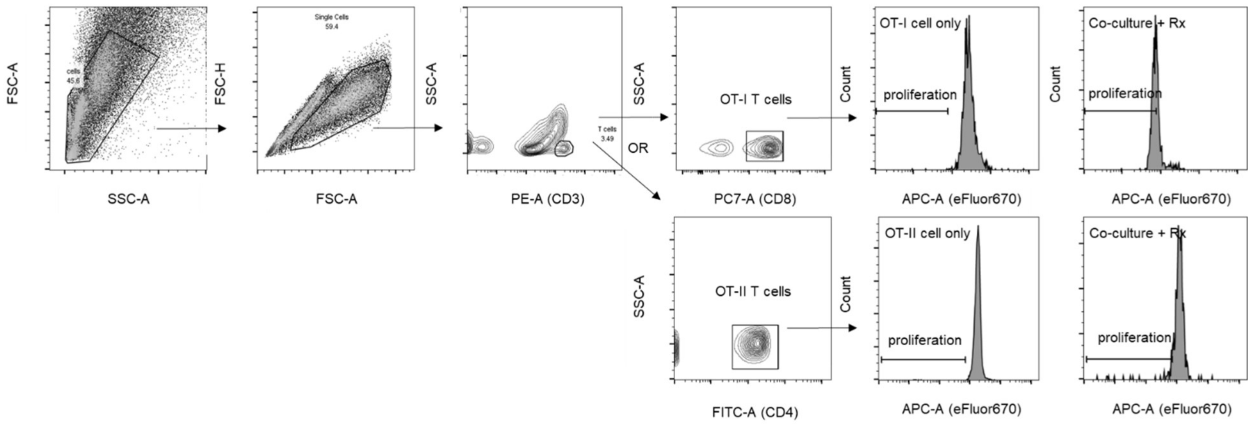

Gating strategy used for OT-I and OT-II proliferation. Gating strategy to identify fraction of proliferating T cells in response to co-culture with drug treated tumor and BMDM cells, used in figures 3h-i, and supplemental figure 11a.

Supplementary Figure 18 (continued): Gating strategies for Flow cytometry experiments.

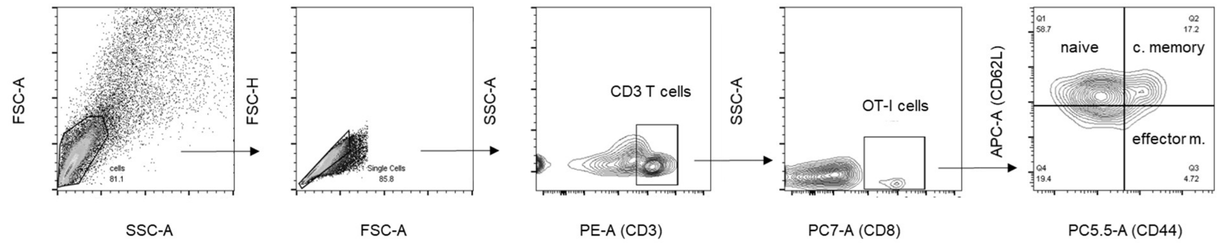

Gating strategy used for OT-I T cell maturation. Gating strategy to identify fraction of OT-I T cells that have matured from a naïve phenotype ($CD62L^{hi}CD44^{lo}$) towards either a central memory ($CD62L^{hi}CD44^{hi}$) or effector memory ($CD62L^{lo}CD44^{hi}$) phenotype in response to co-culture with drug treated tumor and BMDM cells, used in supplemental figure 11b.

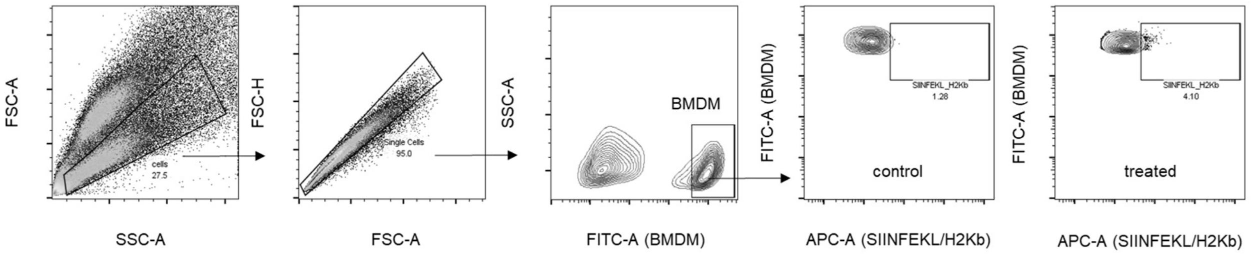

Gating strategy used for antigen presentation. Gating strategy to identify fraction of BMDM cells that express MHCI bound OVA protein (SIINFEKL/H2Kb) in response to co-culture with drug treated tumor cells, used in figures 3f-g.
