# Supplementary material for: Therapeutic modulation of phagocytosis in glioblastoma can activate both innate and adaptive antitumour immunity
Source: Nat Commun. 2020 Mar 20;11:1508. doi: 10.1038/s41467-020-15129-8 (PMC7083893; doi:10.1038/s41467-020-15129-8)
Supplement: Supplementary file 3 — Reporting Summary [file 41467_2020_15129_MOESM3_ESM.pdf]

nature research

Corresponding author(s): Betty Y.S. Kim, Wen Jiang

Last updated by author(s): Jan 22, 2020

Reporting Summary

Nature Research wishes to improve the reproducibility of the work that we publish. This form provides structure for consistency and transparency in reporting. For further information on Nature Research policies, see Authors & Referees and the Editorial Policy Checklist .

Statistics

For all statistical analyses, confirm that the following items are present in the figure legend, table legend, main text, or Methods section.

n/a	Confirmed	
☐	☒	The exact sample size (n) for each experimental group/condition, given as a discrete number and unit of measurement
☐	☒	A statement on whether measurements were taken from distinct samples or whether the same sample was measured repeatedly
☐	☒	The statistical test(s) used AND whether they are one- or two-sided<br>*Only common tests should be described solely by name; describe more complex techniques in the Methods section.*
☒	☐	A description of all covariates tested
☐	☒	A description of any assumptions or corrections, such as tests of normality and adjustment for multiple comparisons
☐	☒	A full description of the statistical parameters including central tendency (e.g. means) or other basic estimates (e.g. regression coefficient) AND variation (e.g. standard deviation) or associated estimates of uncertainty (e.g. confidence intervals)
☐	☒	For null hypothesis testing, the test statistic (e.g. F, t, r) with confidence intervals, effect sizes, degrees of freedom and P value noted<br>*Give P values as exact values whenever suitable.*
☒	☐	For Bayesian analysis, information on the choice of priors and Markov chain Monte Carlo settings
☒	☐	For hierarchical and complex designs, identification of the appropriate level for tests and full reporting of outcomes
☒	☐	Estimates of effect sizes (e.g. Cohen's d, Pearson's r), indicating how they were calculated

Our web collection on statistics for biologists contains articles on many of the points above.

Software and code

Policy information about availability of computer code

Data collection	Microsoft Office Excel, GraphPad Prism, FlowJo, Powerpoint, Keynote
Data analysis	Statistical analyses were performed on Graph Pad Prism. All flow cytometry data were analyzed on FlowJo Software. Fluorescence imaging was analyzed using ImageJ.

For manuscripts utilizing custom algorithms or software that are central to the research but not yet described in published literature, software must be made available to editors/reviewers. We strongly encourage code deposition in a community repository (e.g. GitHub). See the Nature Research guidelines for submitting code & software for further information.

Data

Policy information about availability of data

All manuscripts must include a data availability statement. This statement should provide the following information, where applicable:

- Accession codes, unique identifiers, or web links for publicly available datasets
- A list of figures that have associated raw data
- A description of any restrictions on data availability

All data is presented in the figures and supplemental figures. All source data that was used to generate the figures is provided in the Source Data file.

Field-specific reporting

Please select the one below that is the best fit for your research. If you are not sure, read the appropriate sections before making your selection.

☒ Life sciences ☐ Behavioural & social sciences ☐ Ecological, evolutionary & environmental sciences

Life sciences study design

All studies must disclose on these points even when the disclosure is negative.

Sample size	No effect size was predetermined. The sample sizes were kept consistent with previously published study where in vitro studies were repeated at least three times independently and in the in vivo experiments 4-6 mice per group were used.
Data exclusions	No data were excluded from final analyses.
Replication	All experiments were repeated and findings were consistent.
Randomization	Randomization was used to divide up the animals for in vivo treatment study.
Blinding	No blinding was employed as the researcher performing the treatment was also responsible for the analysis.

Reporting for specific materials, systems and methods

We require information from authors about some types of materials, experimental systems and methods used in many studies. Here, indicate whether each material, system or method listed is relevant to your study. If you are not sure if a list item applies to your research, read the appropriate section before selecting a response.

Materials & experimental systems

n/a	Involved in the study	
☐	☒	Antibodies
☐	☒	Eukaryotic cell lines
☒	☐	Palaeontology
☐	☒	Animals and other organisms
☐	☒	Human research participants
☒	☐	Clinical data

Methods

n/a	Involved in the study	
☒	☐	ChIP-seq
☐	☒	Flow cytometry
☒	☐	MRI-based neuroimaging

Antibodies

Antibodies used

From BioXCell:
Mouse anti-CD47 blocking antibody (clone MIAP-410), Cat# BE0283
Human anti-CD47 blocking antibody (clone B6H12), Cat# BE0019-1
Mouse anti-PD1 antibody (clone 29F.1A12), Cat# BP0273
Mouse anti-CD8 depleting antibody (clone YTS 169.4), Cat# BP0117

From Cell Signaling:
BiP (Clone C50B12; Cell Signaling), Cat# 3177 (WB, 1:1000)
pEIF2a-ser51 (Clone D9G8, Cell Signaling), Cat# 3398 (WB, 1:500)
EIF2a (Clone D7D3, Cell Signaling), Cat# 5324 (WB, 1:1000)
CHOP (Clone L63F7; Cell Signaling), Cat# 2895 (WB, 1:1000)
β-actin (Clone 13E5; Cell Signaling), Cat# 4970 (WB, 1:2000)
pIRF3-S396 (Clone D6O1M; Cell Signaling), Cat# 29047 (WB, 1:500, IF, 1:50)
STING (Clone D2P2F; Cell Signaling), Cat# 13647 (WB, 1:500)
cGAS (Clone D3O8O; Cell Signaling), Cat# 31659 (WB, 1:500)
IKKα (Clone 3G12; Cell Signaling), Cat# 11930 (WB, 1:1000)
IKKβ (Clone D30C6; Cell Signaling), Cat# 8943 (WB, 1:1000)
p-IKKα/β-ser176/180 (Clone 16A6; Cell Signaling), Cat# 2697 (WB, 1:1000)
p-P65-ser536 (Clone 93H1; Cell Signaling), Cat# 3033 (WB, 1:1000; IHC, 1:500)
IκBα (Clone L35A5; Cell Signaling), Cat# 4814 (WB, 1:1000)
p-IκBα-ser32 (Clone 14D4; Cell Signaling), Cat# 2859 (WB, 1:1000)
P65 (Clone D14E12; Cell Signaling), Cat# 8242 (WB, 1:2000)
PD-L1 (clone D5V3B; Cell Signaling), Cat# 64988 (IHC, 1:100)
PD-1 (clone D7D5W; Cell Signaling), Cat# 84651 (IHC, 1:800)

From ThermoFisher:
pSTING-S366 (pAb; ThermoFisher), Cat# PA5-105674 (WB, 1:500)
pIRF3-S385 (pAb; ThermoFisher), Cat# PA5-36775 (WB, 1:500)

IRF3 (pAb; ThermoFisher), Cat# PA5-87506 (WB, 1:500 IHC, 1:100)
SIINFEKL peptide bound to H2Kb (Clone 25-D1.16, Invitrogen), Cat# 14-5743-82 (FC, 0.25ug/10^6 cells; IF, 1:100)
anti-hamster AF647 (ThermoFisher), cat# A-21451 (IF, 1:2000)
anti-rat AF586 (ThermoFisher), cat# A-11077 (IF, 1:2000)
anti-rabbit AF488 (ThermoFisher), cat# A-11034 (IF, 1:2000)
CD4 (clone 4SM95; eBioscience), cat# 14-9766-82 (IHC, 1:100)
CD8 (clone 4SM15; eBioscience), cat# 14-0808-82 (IHC, 1:50)
CD47 (clone B6H12), cat# 14-0479-82 (IHC, 1:50)
CD45 (clone 30-F11, ThermoFisher), #14-0451-82 (IF, 1:100)
CD3 (clone 145-2C11, ThermoFisher), #14-0031-82 (IF, 1:50)

From Abcam:
MGMT (pAb; abcam), cat# ab108630 (WB, 1:500; IHC, 1:100)
CD47 (clone B6H12.2, abcam), cat# ab134484 (FC, 5ul/10^6 cells)
F4/80 (clone SP115; abcam), cat# ab111101 (IHC, 1:500)
Iba1 (pAb; abcam), cat# ab5076 (IHC, 1:2000)

From Bioss:
IFNγ (pAb; Bioss), cat# BS-0480R (IHC, 1:50)

From Biolegend:
CD47 (clone MIAP301; BioLegend), cat# 127507 (FC, 0.5ug/10^6 cells)
CD45 (clone 30-F11, BioLegend), cat# 103121 (FC, 0.25ug/10^6 cells)
CD3 (clone 17A2; BioLegend), cat# 100201 (FC, 0.25ug/10^6 cells)
CD4 (GK1.5; BioLegend), cat# 100425 (FC, 0.25ug/10^6 cells)
CD8 (53-6.7; BioLegend), cat# 100727 (FC, 0.25ug/10^6 cells)
FoxP3 (clone MF-14; BioLegend), cat# 126401 (FC, 0.25ug/10^6 cells)
CD25 (clone 3C7; BioLegen), cat# 101903 (FC, 0.1ug/10^6 cells)
IFNγ (clone XMG1.2; BioLegend), cat# 505815 (FC, 0.25ug/10^6 cells)
CD11b (clone M1/70; BioLegend), cat# 101219 (FC, 0.25ug/10^6 cells)
CD11c (clone N418; BioLegend), cat# 117301 (FC, 0.5ug/10^6 cells)
MHC II (clone M5/114.15.2; BioLegend), cat# 107615 (FC, 0.25ug/10^6 cells)
F4/80 (clone BM8; BioLegend), cat# 123119 (FC, 0.5ug/10^6 cells)
Gr1 (Clone RB6-8C5; BioLegend), cat# 108419 (FC, 0.25ug/10^6 cells)
CX3CR1(clone SA011F11, BioLegend), Cat# 149036 (FC, 0.25 ug/10^6 cells)
P2X7R (clone 1F11, BioLegend), Cat# 148708 (FC, 0.8ug/10^6 cells)

From US Biological:
Calreticulin (pAb; US Biological), cat# 033154 (FC, 1uL/10^6 cells)

Validation: The antibody was verified by the supplier and has been quality tested.

Eukaryotic cell lines

Policy information about cell lines

Cell line source(s): LN229, U87, SWI-1783, G112, T98G, TP365, U251, SWI-008, U118, THP-1, U138 and D32 are from ATCC

Authentication: Cell authentication by the vendor.

Mycoplasma contamination: All cell lines were tested negative for mycoplasma contamination.

Commonly misidentified lines (See ICLAC register): No misidentified cell lines used in the study.

Animals and other organisms

Policy information about studies involving animals; ARRIVE guidelines recommended for reporting animal research

Laboratory animals: C57BL/6, Tmem173gt, OT-I, OT-II, CCr2(RFP)Cx3cr1(GFP) were purchased from the Jax Laboratory.

Wild animals: No wild animals used.

Field-collected samples: No field samples collected.

Ethics oversight	All animal experiments were carried out according to approved IACUC protocols of the Mayo Clinic, University of Florida, and MD Anderson Cancer Center.

Note that full information on the approval of the study protocol must also be provided in the manuscript.

Human research participants

Policy information about studies involving human research participants

Population characteristics	Human GBM and normal brain tissue samples were obtained with informed consent after approval of IRB of Mayo Clinic Jacksonville (IRB Number: 18-009866). No identification information were released with the samples.
Recruitment	Patient with diagnosis of GBM and other non-malignant CNS pathologies were consented for tissue storage and analysis.
Ethics oversight	Use of patient sample was approved by the Institutional Review Board (IRB Number: 18-009866).

Note that full information on the approval of the study protocol must also be provided in the manuscript.

Flow Cytometry

Plots

Confirm that:

- [x] The axis labels state the marker and fluorochrome used (e.g. CD4-FITC).
- [x] The axis scales are clearly visible. Include numbers along axes only for bottom left plot of group (a 'group' is an analysis of identical markers).
- [x] All plots are contour plots with outliers or pseudocolor plots.
- [x] A numerical value for number of cells or percentage (with statistics) is provided.

Methodology

Sample preparation	For surface antigen staining, tumor and/or APCs were collected, and washed 2x with FACS buffer (PBS containing 1% BSA and 2mM EDTA). Fc blocking was performed using TruStain FcX (BioLegend), followed by fluorescent labeled primary antibody staining for 1 h on ice. Cells were washed with FACS buffer, and viability stain was added just prior to analysis. For intracellular antigen labeling, cells were washed 2x with FACS buffer, and labeled with LIVE/DEAD fixable dye per manufacturer protocol (ThermoFisher). Cell fixation, permeation, Fc Block (BioLegend), and staining was performed using the PerFix-nc Kit (Beckman Coulter) prior to analysis.
Instrument	CytoFLEX flow cytometer (Beckman Coulter)
Software	FlowJo
Cell population abundance	No sorting was performed.
Gating strategy	Gating strategy for immune cell populations are included in the figures or in Supplementary Information.

- [x] Tick this box to confirm that a figure exemplifying the gating strategy is provided in the Supplementary Information.
